# Total Alkaloids of Rhizoma Corydalis regulates gut microbiota and restores gut immune barrier to ameliorate cognitive dysfunction in diabetic rats

**DOI:** 10.3389/fmicb.2024.1456406

**Published:** 2024-12-02

**Authors:** Yazhi Qi, Jun Li, Ya Tang, Rui Cao, Yishu Gao, Qiang Xu, Yusheng Han

**Affiliations:** ^1^Basic Medical College, Heilongjiang University of Chinese Medicine, Harbin, China; ^2^Jiamusi Campus, Heilongjiang University of Chinese Medicine, Jiamusi, China

**Keywords:** Total Alkaloids of Rhizoma Corydalis, gut microbiota, cognitive dysfunction, diabetes, rats

## Abstract

**Background and objectives:**

Given the widespread dysbiosis of gut microbiota in patients with T2DM, it has been found that the microbiota-gut-brain axis plays an influential regulatory role in diabetic cognitive dysfunction, and improving gut dysbiosis may be a potential strategy for treating diabetic cognitive dysfunction. Total Alkaloids of Rhizoma Corydalis (TAC) is the main active component extracted from Rhizoma Corydalis. Pharmacological studies have demonstrated its significant pharmacological effects on the cardiovascular and cerebrovascular systems, and berberine, the main component of TAC, has a certain regulatory effect on gut microbiota.

**Materials and methods:**

Rats were randomly divided into Control group, Model group, TAC-low group, TAC-mid group and TAC-high group. Cognitive function of diabetic rats was evaluated through behavioral testing using the Morris water maze experiment. The relative abundance of gut bacteria in rat feces was determined via 16S rRNA analysis. IHC and Western blot techniques were employed to assess IL-22, IL-23, Reg3g, ZO-1, occludin 1 expression in the colon tissue; GPX4, xCT, NLRP3, Caspase-1 p20, GSDMD-N were detected in the hippocampus.

**Results:**

The cognitive function of diabetic rats decreased significantly. TAC demonstrated a significant reduction in inflammatory factors in serum, hippocampus, and colon, thus alleviating inflammation. Additionally, it effectively decreased ferroptosis induced by NLRP3 and reduced pathological damage in the hippocampus of diabetic rats. After treatment, the differential microbiota such as *Lachnoclotridium* and *Bacteroides*. TAC improved gut barrier permeability and integrity in rats while remodeling gut mucosal homeostasis. Moreover, pyroptosis and ferroptosis caused by the inflammatory cascade in the rat hippocampus were also significantly inhibited.

**Conclusion:**

The combination of high lipid and high glucose with STZ can result in gut microbiota disturbance, damage gut immune barrier, decreased gut mucosal permeability and integrity, aggravated gut inflammation, further spread inflammatory factors to brain tissue, cause inflammatory cascade reaction of encephalopathy, and ultimately resulting in neuronal ferroptosis and cognitive dysfunction in diabetes mellitus. Our study suggests that TAC may regulate gut microbiota, restore gut immune homeostasis, improve gut barrier permeability and integrity, inhibit brain tissue inflammatory cascade, reduce neuronal ferroptosis, and thus improve diabetes. This provides new targets for its treatment strategy.

## 1 Introduction

The incidence of type 2 diabetes mellitus (T2DM) is gradually increasing due to the aging population and the rise in obesity. Cognitive dysfunction is one of the most serious complications of diabetes (Xue et al., 2019), and the literature has shown that patients with T2DM have a 1.5–2.5 times greater risk of developing dementia than the general population (Biessels et al., 2006). Experimental studies have found that the etiology and progression of T2DM to diabetes cognitive dysfunction may be significantly mediated by the gut microbiota (Huang et al., 2023). The connection between the gut microbiota and brain is tightly intertwined through a bidirectional communication system called the microbiota-gut-brain axis, in which inflammation plays an essential role (Agirman et al., 2021). The occurrence and progression of diabetes are accompanied by the disorder of gut microbiota (Liu et al., 2020). Consumption of a high-fat and high-sugar diet can disrupt the intestinal immune barrier through its effects on gut microbiota, leading to chronic low-level activation of the inflammatory system. This inflammation may eventually propagate from peripheral tissues to the brain, triggering an inflammatory cascade in the brain and resulting in cognitive dysfunction (Solas et al., 2017). In this process, we have discovered a close association between chronic inflammation and ferroptosis, which is a newly identified form of cell death characterized by intracellular iron accumulation and lipid peroxidation. Recent studies have shown that ferroptosis is related to a variety of neurological diseases, including cognitive dysfunction and neurodegenerative diseases (Ratan, 2020). In the inflammatory environment, the imbalance of iron metabolism can lead to neuronal ferroptosis (Lee and Hyun, 2023), and studies have proved that NLRP3, the classical pathway of pyroptosis, can promote the occurrence and development of ferroptosis (Li Z. et al., 2023). Therefore, it is hypothesized that gut microbiota may compromise the immune barrier, triggering an inflammatory cascade that activates the NLRP3 pyroptosis signaling pathway. This activation could promote neuronal ferroptosis and result in cognitive dysfunction in diabetic rats (Bi et al., 2021).

The treatment of diabetic cognitive dysfunction in modern medicine mainly refers to the clinical medication of MCI and AD, but it has not shown obvious clinical efficacy (Xiong et al., 2021). Commonly used hypoglycemic drugs such as metformin and glibenclamide have limited effects on improving diabetic cognitive dysfunction (Launer et al., 2011). Rhizoma Corydalis is a dried rhizome derived from Papaveraceae and is a traditional Chinese medicine commonly used in clinical practice, especially for cardiovascular and nervous systems (Xu et al., 2021). Tetrahydropalma is the principal component of Total Alkaloids of Rhizoma Corydalis, with a content of 50%. Other subordinate components include Corydaline, Protopine, Corydalis, Corydalis H, Glaucine and Palmatine, which are present in a total content of 30% (Zhang et al., 2016). The previous research conducted by our research group has demonstrated that TAC exhibits pharmacological effects such as anti-inflammatory properties, inhibition of apoptosis and pyroptosis, which have the potential to significantly improve conditions such as epilepsy (Qi et al., 2023), cerebral ischemia (Li J. et al., 2023), etc.; A number of *in vivo* and *in vitro* related experiments have also verified that TAC has anti-depressant and anti-anxiety pharmacological effects in addition to anti-inflammatory, antibacterial, and analgesic effects (Alhassen et al., 2021); The main component of the TAC, berberine, can not only promote insulin release (Zhao et al., 2021) and reduce insulin resistance (Zhang et al., 2010), but also improve gut microbiota imbalance, and relieve anxiety (Fang et al., 2021). Collectively, these findings indicate promising prospects for further research and development of TAC.

In this study, T2DM rats were fed with high-lipid diet for 16 weeks and received intraperitoneal injection of STZ. Morris water maze test was used to evaluate cognitive dysfunction, 16S rRNA was used to detect the changes of gut microbiota structure, IHC and Western blot were used to detect gut immune barrier function, brain inflammation and ferroptosis-related proteins. Whether TAC can improve cognitive dysfunction by regulating gut microbiota to inhibit ferroptosis in diabetic rats was observed.

## 2 Materials and methods

### 2.1 Animals and medicines

Eight-week-old SPF male SD rats, weighing 180 ± 20 g, were purchased from Liaoning Changsheng Biotechnology Co., Ltd. The experimental animal license number is SCXK (Liao) 2020-0001. The housing conditions maintained a temperature range of 20–25°C and relative humidity at 50–60%, with *ad libitum* access to food and water. Animal experiments were approved by the Experimental Animal Welfare Ethics Committee of Heilongjiang University of Traditional Chinese Medicine, project number: 2023112907. TAC was prepared in accordance with the previous methods of the research group. Specifically, the extraction method was acid water percolation. The percolation endpoint was determined by the detection of alkaloid precipitation reagents. Tetrahydropalmatine was used as the standard substance, and the content of TAC was determined by the acid dye colorimetric method. Considering various factors, it was determined that the optimal percolation volume was 12 times the mass of the medicinal materials, resulting in an extraction rate of TAC reaching 85% (Wang and Tian, 2015).

### 2.2 Laboratory apparatus

Morris water maze behavioral analysis system was provided by Shanghai Xinruan Information Technology Co., Ltd.; DNM -9602 microplate reader was obtained from Beijing Perlong New Technology Co., Ltd.; Upright fluorescence microscope FR-4A is from Shanghai Optical Instrument Factory; YD-1508B tissue slicer is manufactured by Zhejiang Jinhua Yidi Medical Equipment Factory; MoticBA400 microscopic photography and imaging system is a product of Motic Inc., United States; iCEN-24R tabletop high-speed refrigerated centrifuge was purchased from Hangzhou Aosheng Instrument Co., Ltd.; Electrophoresis equipment including Tanon EPS-300 electrophoresis apparatus, Tanon VE-186 electrophoresis tank, and Tanon VE-180B transfer tank were supplied by Shanghai Tianneng Technology Co., Ltd. Gel imaging was performed using the Tanon-4600 gel imaging system also from Shanghai Tianneng Technology Co., Ltd. Gut microbiota sequencing was entrusted to Shanghai Majorbio Biomedical Technology Co., Ltd.

### 2.3 Experimental design and administration

Grouping was conducted using a completely random method. The 35 rats were numbered from 1 to 35. Thirty five random numbers were randomly obtained from the random number table in the same direction. The random numbers were divided by the number of groups (5) to obtain the remainders. The groups were constantly adjusted until there were 7 rats in each group. Four randomly selected groups of rats were fed a high-fat diet (78.8% basal diet +15% lard +1% cholesterol +5% sucrose +0.2% sodium cholate) for 16 weeks in combination with two intraperitoneal injections of 30 mg·kg^−1^ streptozotocin to establish a T2DM rat model, with a 1-week interval. Seven days after the last STZ injection, fasting blood glucose greater than 16.5 mmol·L^−1^ indicated that a total of 24 rat models of diabetes were successfully established. The 4 groups of rats were randomly divided into the model group and the low-, medium-, and high-dose TAC groups. One healthy rat was randomly excluded from the control group, with 6 rats in each group. The administration groups were gavaged with the corresponding doses of drugs (low, medium, and high doses were 7, 11, and 14 g·kg^−1^, respectively) for 4 consecutive weeks.

### 2.4 Index collection and detection

#### 2.4.1 Morris water maze experiment

Each group of rats were tested with the Morris water maze experiment. (1) Place navigation test: Rats in each group were positioned facing the pool wall at four different quadrant markers, with a 1-h interval between each training session. The latency period, which is the time taken for the rats to locate the platform within 60 s, was recorded. For those rats that did not find the platform within 60 s, they were re-placed on the platform for 10 s before being removed from the maze, and their latency period was recorded as 60 s. Taking the average of 4 latency period as the final result, this training was conducted continuously for 4 days. (2) Spatial probe test: On the fifth day, the platform was removed, and the rats were placed in the water facing the wall of the pool in the opposite quadrant of the platform; their latent time and number of times crossing over where it had been located were then recorded.

#### 2.4.2 Fasting blood glucose test

For the FBG test, animals were fasted for 9 h and tested on blood glucose test strips measured by a glucose meter before tail blood was collected (Roche, United States). FBG was measured weekly before STZ injection, after STZ injection (weeks 1, 2), and during TAC treatment (weeks 1, 2, 3, 4). Each blood glucose measurement was performed three times for each animal at every time point, and the average value was recorded.

#### 2.4.3 16S rRNA test

Bacterial genomic DNA was extracted from feces using a reagent kit, and a gene library was constructed. OTU clustering of sequences at 97% similarity was performed using bioinformatics methods with USEARCH software. The sequences were then annotated for taxonomic classification against the Silva 16S rRNA gene database (v138) with a confidence threshold of 70%, and the community composition of each sample was statistically analyzed at different taxonomic levels. Mothur was utilized for calculating Alpha diversity indices such as Simpson, Shannon, and for conducting Wilcoxon rank-sum tests to assess inter-group differences in Alpha diversity. Beta diversity analysis was employed to evaluate the similarity and dissimilarity of samples from different groups in terms of community structure, utilizing multivariate statistical analysis methods such as PcoA to analyze the results. The Kruskal-Wallis rank sum test was employed for multi-group difference tests to assess the intergroup differences among the three groups. Lefse multi-level discriminant analysis of species difference (multi-level: phylum, class, order, family, genus) was used to test the difference at multiple levels, analyze the multi-level differential species and screen the differential microbiota. The heat map of the correlation coefficient between dominant microorganisms and pharmacodynamic indexes was drawn, and the key genera and species affecting diabetic cognitive dysfunction were screened out.

#### 2.4.4 Detection of insulin in rat serum, and the contents of IL-1β, IL-18, IL-6, and TNF-α in serum, hippocampus and colon using enzyme-linked immunosorbent assay

The blood, colon and tissue supernatant of rats in each group were extracted and detected using ELISA kits for IL-1β, IL-18, IL-6, and TNF-α according to the manufacturer’s instructions (Andy gene, Beijing, China).

#### 2.4.5 Observation of pathological changes in hippocampus and colon of rats in each group using hematoxylin-eosin staining

Before performing HE staining, the brain tissue and colon of rats from each group were fixed in 4% paraformaldehyde solution for 24 h, then embedded in paraffin wax, and then cut into slices with a thickness of 5 μm. The pathological changes in hippocampus and colon of rats from each group were then observed under microscope.

#### 2.4.6 PAS staining of rat colon tissue

Paraffin sections were routinely dehydrated, stained with periodic acid Schiff (PAS) at room temperature for 30 min, counterstained with hematoxylin, dehydrated, and then sealed with neutral gum; The changes of glycogen content in colon tissue of each group were observed under microscope, and the statistical analysis was performed using ImageJ.

#### 2.4.7 Assessment of ferroptosis

Iron deposition, iron content, and SOD, MDA, 4-HNE, GSH, and GSH-PX levels were measured in rat brain hippocampal neurons to assess ferroptosis. Iron deposits in the brain were determined by Perls staining using the Perls staining kit (LEAGENE Biotechnology Co., Ltd., Anhui, China) according to the instructions. Iron content in serum and brain tissue of rats was measured using serum iron assay kit (Nanjing Jiancheng Bioengineering Institute, Nanjing, China) and tissue iron assay kit (Nanjing Jiancheng Bioengineering Institute, Nanjing, China), respectively. The contents of SOD, MDA, GSH, GSH-PX, and 4-HNE were determined by SOD kit (Nanjing Jiancheng Bioengineering Institute, Nanjing, China), MDA kit (Nanjing Jiancheng Bioengineering Institute, Nanjing, China), GSH kit (Nanjing Jiancheng, Nanjing, China), GSH-PX kit (Nanjing Jiancheng Bioengineering Institute, Nanjing, China), and 4-HNE Elisa kit (Beijing Biotoppeed Technology Co., Ltd., Beijing, China), respectively.

#### 2.4.8 NLRP3, Caspase-1p20, GSDMD-N, GPX4, xCT in hippocampus, IL-22, IL-23, Reg3g, ZO-1, occludin1 protein in colon were detected by immunohistochemistry

Paraffin sections were routinely dewaxed, incubated with 10% H2O2 for 10 min and repaired by microwave; Endogenous enzymes were inactivated and washed with primary antibodies. The primary antibodies used were NLRP3 (1: 125, Servicebio, Wuhan, China), Caspase-1p20 (1: 100, Bioss, Beijing, China), GSDMD-N (1: 125, Affinity Biosciences, Jiangsu, China), GPX4 (1: 75, FineTest, Wuhan, China), xCT (1:50, ABclonal, Wuhan, China), IL-22 (1: 150, Wanleibio, Shenyang, China), IL-23 (1: 125, Bioss, Beijing, China), Reg3g (1: 125, Bioss, Beijing, China), ZO-1 (1: 150, Wanleibio, Shenyang, China), occludin1 (1: 150, Wanleibio, Shenyang, China), with a ambient temperature of 4°C overnight. Subsequently, the secondary antibody was added dropwise and incubated at 37°C for 30 min; The nuclei were counterstained with hematoxylin after DAB development; Seal after dehydration and transparency. Six cases from each group were selected for detection, and images were collected under a microscope at 200 fold magnification of the Motic3000 microphotography imaging system. Three different fields were randomly selected in each slice, and analyzed using Image-pro plus6.0 image analysis software to represent protein expression level as mean integrated optical density (IOD).

#### 2.4.9 Western blot was used to detect NLRP3, Caspase-1p20, GSDMD-N, GPX4, xCT in hippocampus, IL-22, IL-23, Reg3g, ZO-1, occludin1 in colon

Proteins were extracted from the hippocampus and colon of rats, denatured, and then loaded into sample wells for electrophoresis separation. Following transfer and blocking for 2 h, primary antibodies (Servicebio, Wuhan, China), Caspase-1p20 (Bioss, Beijing, China), GSDMD-N (Affinity Biosciences, Jiangsu, China), GPX4 (FineTest, Wuhan, China), xCT (ABclonal, Wuhan, China), IL-22 (Wanleibio, Shenyang, China), IL-23 (Bioss, Beijing, China), Reg3g (Bioss, Beijing, China), ZO-1 (Wanleibio, Shenyang, China), occludin1 (Wanleibio, Shenyang, China). All at a dilution ratio of 1:1,000 were incubated overnight. After incubation at room temperature for 1 h, ECL chemiluminescence was developed uniformly. The captured images were analyzed using ImageJ software to calculate the grayscale value ratio of the target protein bands to the internal reference bands in each group to determine the expression level of the target protein.

#### 2.4.10 Statistical treatment

The data were analyzed using SPSS25.0, and normally distributed data were expressed as mean ± SD. One-way ANOVA was used to analyze the experimental data analysis, and the LSD-t was used for pairwise sample tests. A significance level of *p* < 0.05 was considered statistically significant.

## 3 Results

### 3.1 TAC can ameliorate learning and memory impairment in STZ-induced diabetic rats

**Figure 1:**
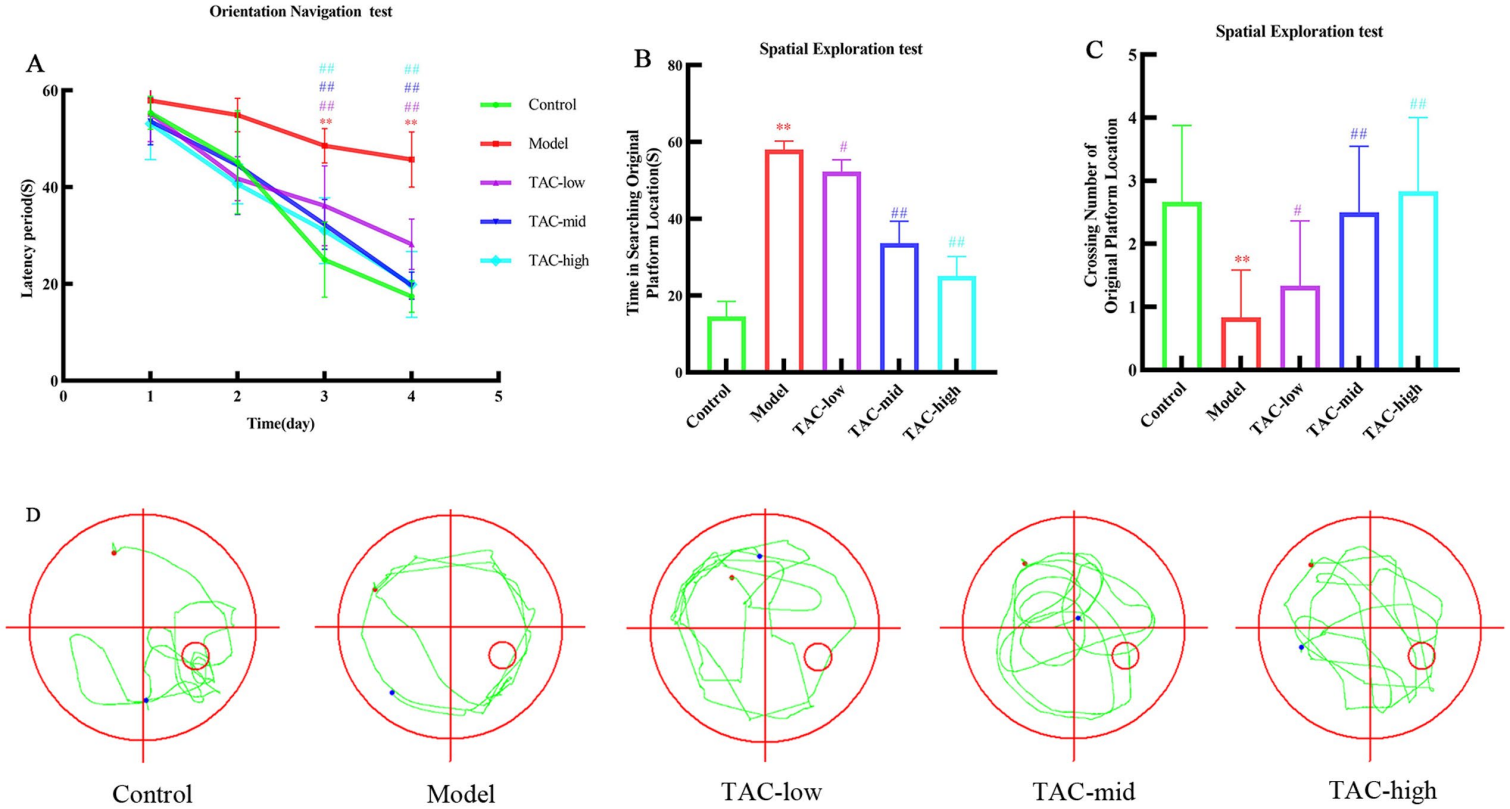
Effect of TAC on learning and memory performance in diabetic rats. **(A)** Escape latency period during the 4-day training of the place navigation test. **(B–D)** In the spatial probe test, rats escaped latency period **(B)**, number of crossings at platform positions **(C)**, representative swimming paths **(D)** mean ± SD. *n* = 6. Statistical analysis was performed using one-way ANOVA **p < 0.01 vs. Control group; ^#^*p* < 0.05 vs. Model Group. ^##^*p* < 0.01 vs. Model Group.

Morris water maze test was used to detect spatial learning and memory of rats in each group. The results showed that over the initial 4 days, as training duration increased, escape latency in locating the platform gradually decreased across all groups. The Model group exhibited a significant increase in escape latency compared to the Control group; however, TAC at various doses led to a significant reduction in escape latency compared to the Model group (Figure 1A). Following removal of the platform, analysis of day 5 crossings indicated a reduced number of platform crossings and significantly prolonged latency period for the Model group compared to the Control group. In contrast, each dose group increased the number of platform crossings and significantly shortened latency period compared to the Model group (Figures 1B,C). According to the judgment of the positioning trajectory map on the fifth day, it was found that the rats in the Model group could not quickly find the quadrant where the platform was located, and rotated around the wall in 4 quadrants. Platforms were easier to find across dose groups, and memory was also significantly improved in rats (Figure 1D). These data suggest that TAC has potential for mitigating diabetes-induced impairments in learning and memory while positively impacting cognitive function.

### 3.2 The changes of body weight, blood glucose and serum insulin in each group

**Figure 2:**
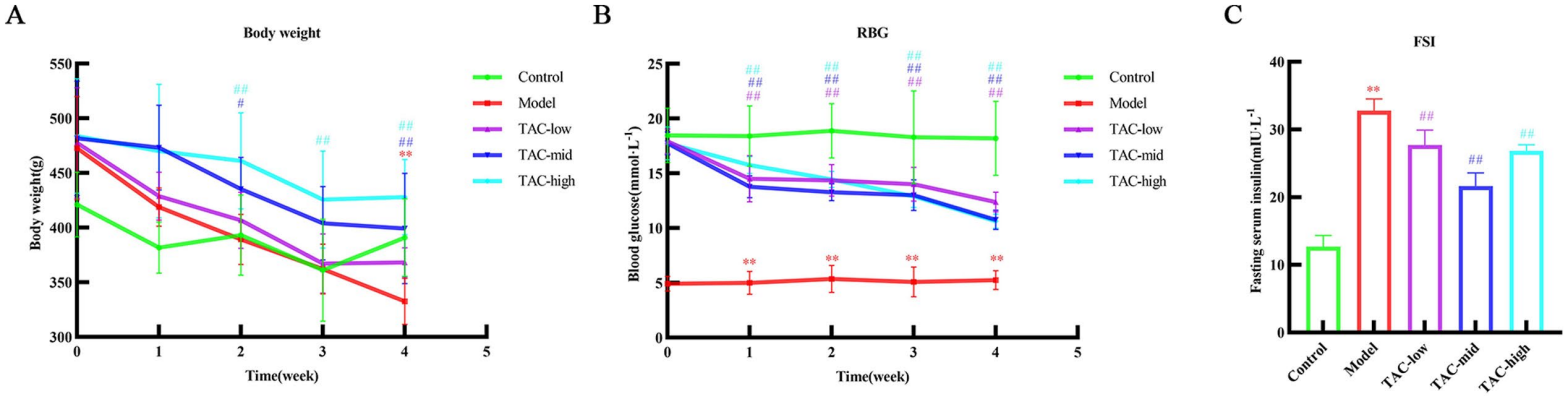
Effect of TAC on body weight, blood glucose and serum insulin in diabetic rats. Body weight **(A)**, blood glucose **(B)**, and serum insulin **(C)** mean ± SD. *n* = 6. Statistical analysis using one-way ANOVA **p < 0.01 vs. Control group; ^#^*p* < 0.05 vs. Model Group. ^##^*p* < 0.01 vs. Model Group.

Body weight (Figure 2A), blood glucose (Figure 2B), and serum insulin (Figure 2C) in rats were measured. Compared with the Control group, there was a significant decrease in body weight and a significant increase in blood glucose and serum insulin levels. In contrast, rats treated with TAC exhibited a significant increase in body weight and a significant decrease in serum insulin and blood glucose levels compared to the Control group.

### 3.3 Effects of TAC on gut microbiota in diabetic rats

**Figure 3:**
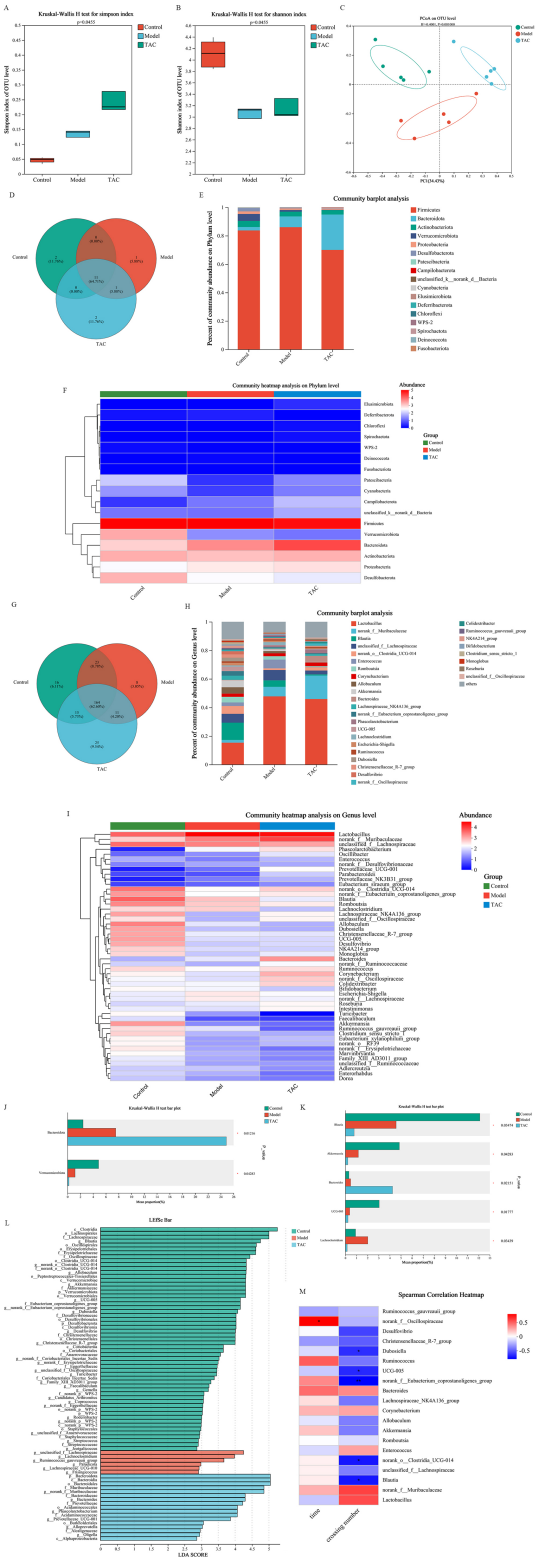
Effect of TAC on alpha and beta diversity indices in colonic microbiota. Alpha diversity was analyzed by Simpson **(A)** and Shannon **(B)**, and beta diversity was analyzed by PCoA **(C)**, with each point representing an individual sample. **(D)** Venn diagram of gut microbiota at the phylum level. **(E,F)** Composition of gut microbiota at the phylum level. **(G)** Venn diagram of gut microbiota at the genus level. **(H,I)** Composition of gut microbiota at the genus level. The **(J,K)** Kruskal-Wallis rank sum test assessed the differences between the three groups. **(L)** Lefse multilevel discriminant analysis of species differences **(M)** Heat map of correlation coefficients between dominant microorganisms and pharmacodynamic indexes. *p < 0.05, **p < 0.01.

Alpha diversity of gut microbiota was analyzed by Simpson and Shannon. Among them, Simpson (Figure 3A) and Shannon (Figure 3B) in the Control group were lower than those in the Model and TAC groups. Furthermore, PCoA (Figure 3C) was employed to assess beta diversity. In this study, there was a clear separation between the 3 groups, suggesting that there may be different compositions of the intestinal flora among these 3 groups. To determine the target differential bacteria, differential bacteria between the 3 groups were evaluated. The Venn diagram results of this study show that at the gate level, there are 11 OTUs in the Control group and TAC group, and 12 OTUs in the Model group and TAC group. At the genus level, there were 179 OTUs in the Control and TAC groups, and 175 OTUs in the Model and TAC groups (Figures 3D,G). We found that *Firmicutes* were the predominant flora. Compared with the Control group, the abundance of *Bacteroidota* increased and the abundance of *Actinobacteriota* decreased in the Model group; The abundance of *Bacteroidota* increased significantly and the abundance of *Firmicutes* decreased in the TAC group compared with the Model group (Figures 3E,H). These are heat maps at the Phylum and Genus levels (Figures 3F,I). The Kruskal-Wallis rank-sum test and Lefse multilevel species differences were used for discriminant analysis. *Bacteroidota* and *Verrucomicrobiota* were found to be significantly different between the three groups at the phylum level by significance analysis (Figures 3J,L); At the genus level, the abundance of *Bacteroides* and *Lachnoclotridium* in the Model group was higher than that in the Control group, and the abundance of *Blautia*, *Akkermansia* and *UCG-005* was significantly reduced. Meanwhile, TAC treatment significantly increased the abundance of *Bacteroides* and decreased the abundance of *Blautia*, *Akkermansia*, *UCG-005*, and *Lachnoclotridium* (Figures 3K,L). The heat map shows the correlation coefficients between the abundance of 20 genera and the water maze latency and the number of platforms crossed in diabetic cognitively dysfunctional rats (Figure 3M). These findings suggest that the intestinal flora is involved in cognitive function in diabetic rats.

### 3.4 TAC attenuates inflammatory response in diabetic rats

**Figure 4:**
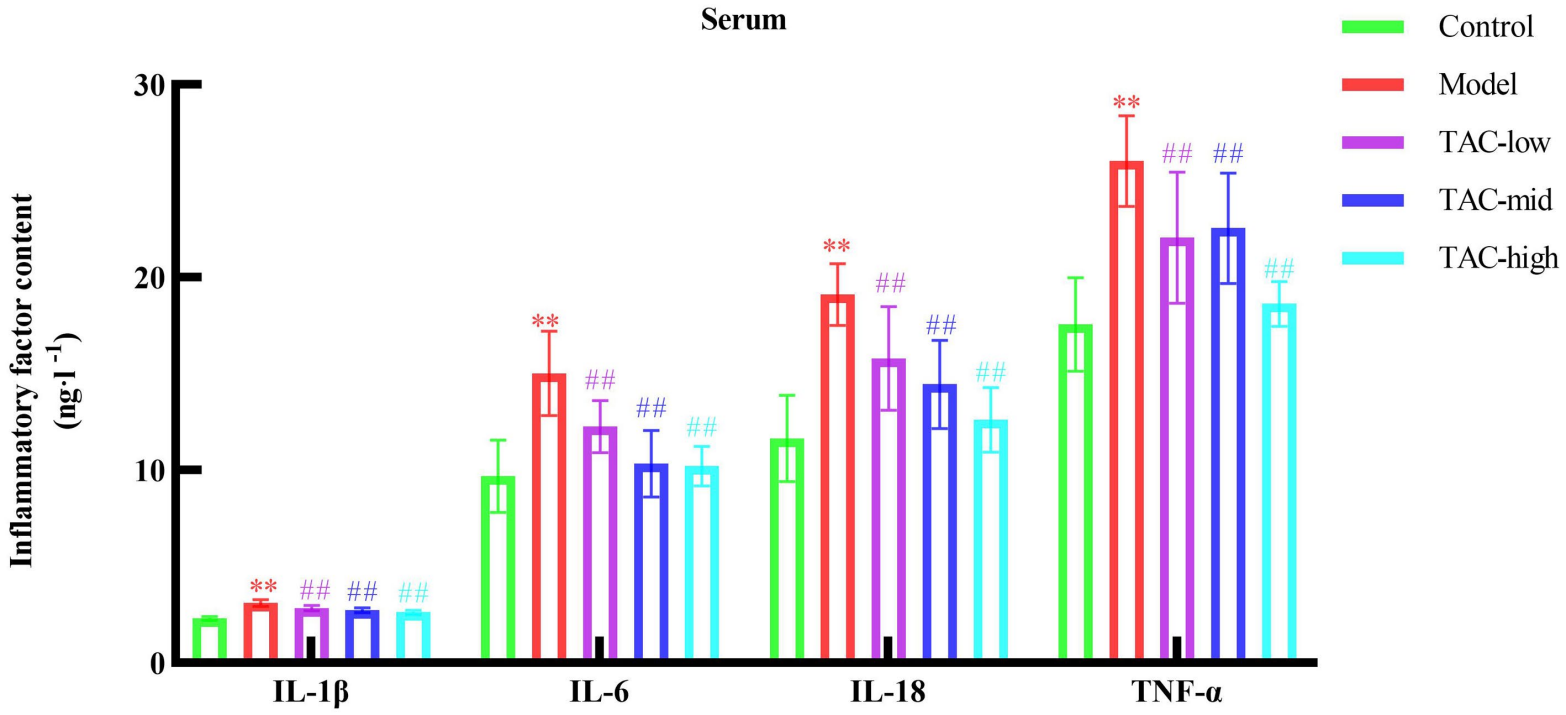
Effect of TAC on inflammatory factors IL-1β, IL-6, IL-18, TNF-α in serum of diabetic rats. Mean ± SD. *n* = 6. Statistical analysis using one-way ANOVA **p < 0.01 vs. Control group; ^##^*p* < 0.01 vs. Model Group.

By detecting the levels of inflammatory factors IL-1β, IL-6, IL-18, and TNF-α in the serum of rats in each group (Figure 4), the results showed that compared with the Control group, the levels of various inflammatory factors in the Model group were increased; Compared with the Model Group, the levels of inflammatory factors in each dose group were reduced. It is demonstrated that TAC can effectively alleviate the systemic chronic inflammatory response caused by diabetes.

### 3.5 TAC can ameliorate the pathological changes of hippocampal and colonic barrier in diabetic rats

**Figure 5:**
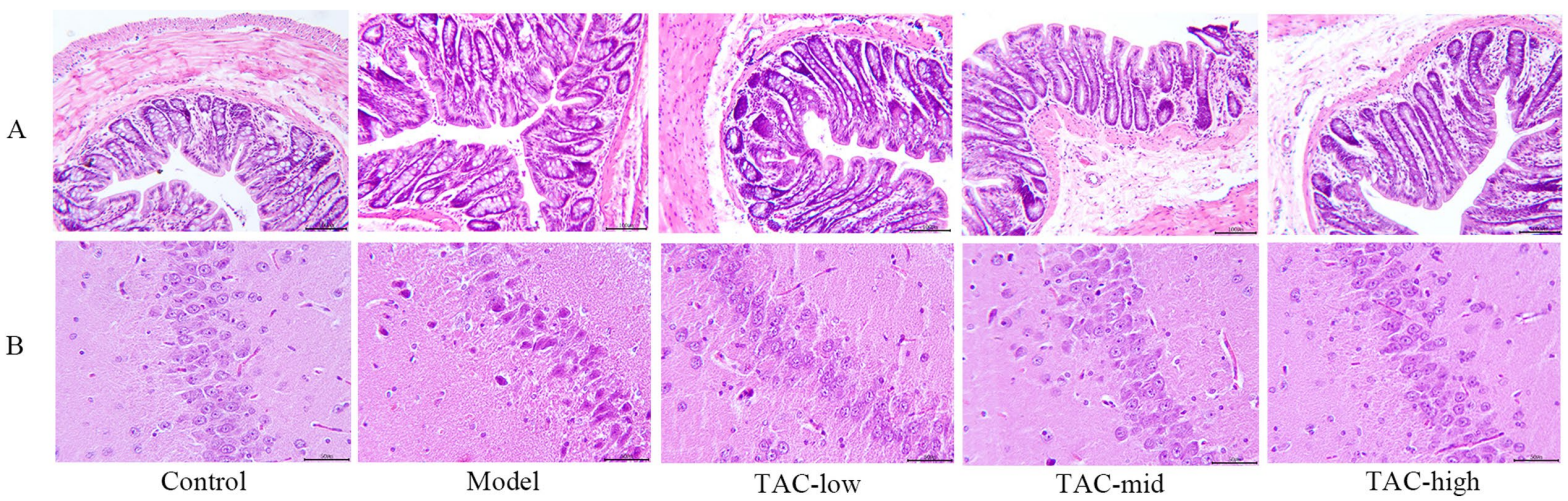
Changes of TAC on colon and hippocampus pathological morphology in diabetic rats. **(A)** HE × 100, scale bar = 50 μm, **(B)** HE × 200, scale bar = 50 μm.

Hematoxylin and eosin (H & E) staining were used to evaluate the hippocampal and colonic pathological changes of diabetic rats before and after TAC treatment. In Control Group, the colonic structure was clear, the gut mucosa arranged regularly, the structure of gut gland cells was normal, the cytoplasm and nucleus stained clearly and evenly, and there was no inflammatory cell infiltration or capillary congestion. In contrast, the Model group showed severe pathological changes in the colonic tissue, gut mucosa was disorganized, gut villi fell off, epithelial cells were deeply stained, the number of goblet cells was reduced, telangiectasia in the submucosa, and inflammatory cells were infiltrated. Notably, TAC treatment relieved colonic epithelial morphology, with significant improvement in the aforementioned pathological changes, increased number of goblet cells, decreased inflammatory cells, and reduced telangiectasia compared to the Model group (Figure 5A). In the Control group, hippocampal tissue had no edema, neurons arranged regularly, the boundaries between structures were clear, and no large number of inflammatory cells were seen. Model group hippocampal tissue structure loose, inflammatory cell infiltration, karyopyknosis significantly increased, microglia significantly increased, vasodilation and congestion obvious. In each dose group, the hippocampal tissue was relatively tidy, the infiltration of inflammatory cells and microglia were significantly reduced, and the vasodilation and congestion were also relatively relieved (Figure 5B).

### 3.6 TAC can regulate the balance of gut immune function and reduce the damage of mucosal barrier

**Figure 6:**
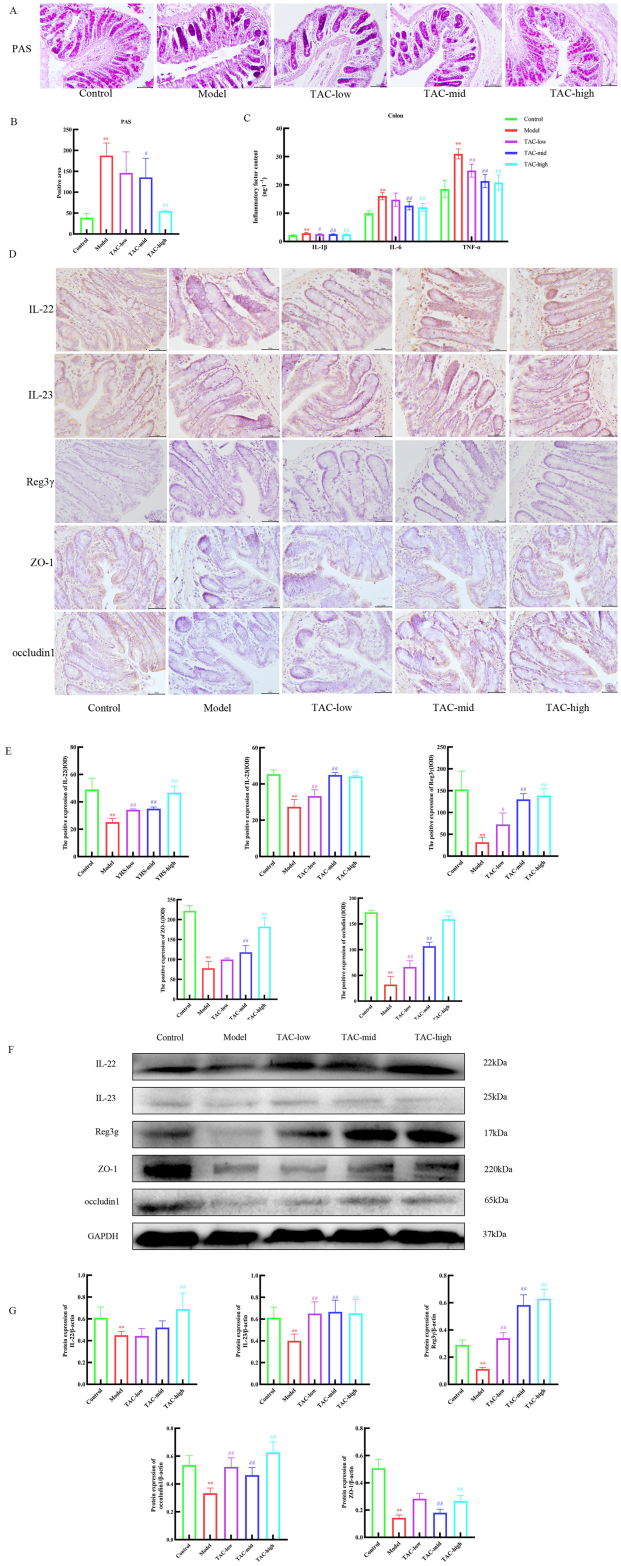
Effect of TAC on colonic immune homeostasis, mucosal barrier in diabetic rats. **(A,B)** Colon PAS staining **(C)** Effect of TAC on inflammatory factors IL-1β, IL-6, TNF-α in the colon of diabetic rats. **(D,E)** Immunohistochemical detection results of IL-22, IL-23, and Reg3γ in the colon. **(F,G)** Western blot results of IL-22, IL-23 and Reg3γ detection in the colon. Mean ± SD. *n* = 3. Statistical analysis using one-way ANOVA **p < 0.01 vs. Control group; ^#^*p* < 0.05 vs. Model Group. ^##^*p* < 0.01 vs. Model Group.

PAS staining results of the colon tissue (Figures 6A,B) showed that the glycogen content of the Model group was significantly increased compared with that of the Control group, and the differences in inflammatory factors such as IL-1β, IL-6, and TNF-α in the colon were also significant. TAC improved these indicators to a certain extent (Figure 6C). IHC and WB results on colonic IL-22, IL-23, and Reg3γ showed that compared with the Control group, the levels of IL-22, IL-23, and Reg3γ in the colon of diabetic rats were significantly reduced, while TAC could increase the expression of IL-22, IL-23, and Reg3γ in the colon of diabetic rats (Figures 6D–G). These results suggest that TAC can inhibit colonic inflammation and restore gut immune balance in diabetic rats. As shown in Figure 6F, ZO-1 and occludin1 expression was significantly reduced in diabetic rats compared to the Control group, indicating impaired gut mucosal barrier. However, compared to the Model group, the TAC-treated group showed a significant increase in ZO-1 and occludin1 protein expression, indicating that TAC is associated with the protective effect of the gut mucosal barrier.

### 3.7 TAC can inhibit inflammatory cascade in brain tissue

**Figure 7:**
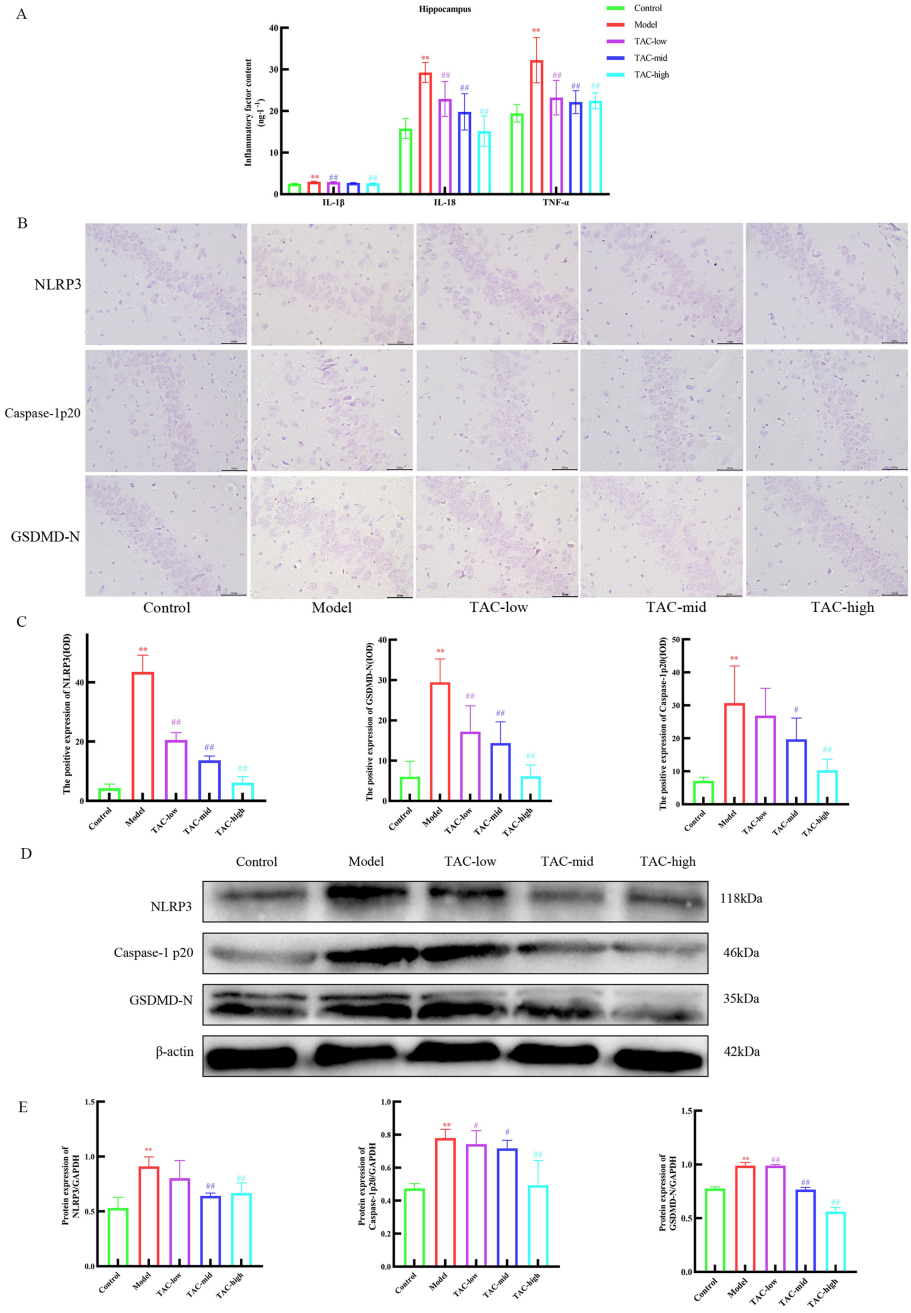
Effect of TAC on the inflammatory cascade in the brain tissue of diabetic rats. **(A)** Effects of TAC on inflammatory factors IL-1β, IL-18, TNF-α in hippocampus of diabetic rats. **(B,C)** Immunohistochemical detection results of NLRP3, Caspase-1p20 and GSDMD-N in hippocampus. **(D,E)** Western blot results of IL-22, IL-23, and Reg3γ in the colon. Mean ± SD. *n* = 3. Statistical analysis using one-way ANOVA **p < 0.01 vs. Control group; ^#^*p* < 0.05 vs. Model Group. ^##^*p* < 0.01 vs. Model Group.

Inflammatory factors such as IL-1β, IL-18, and TNF-α were significantly different in the hippocampus (Figure 7A). The results of IHC and WB detection of NLRP3, Caspase-1p20 and GSDMD-N in the brain (Figures 7B–E) showed that the levels of NLRP3, Caspase-1p20 and GSDMD-N in the hippocampus of diabetic rats were significantly increased compared with Control group, while TAC could reduce the expression of NLRP3, Caspase-1p20 and GSDMD-N in the hippocampus of diabetic rats. It is suggested that TAC can alleviate pyroptosis caused by the inflammatory cascade mediated by the brain-gut axis.

### 3.8 TAC can inhibit ferroptosis in brain tissue

**Figure 8:**
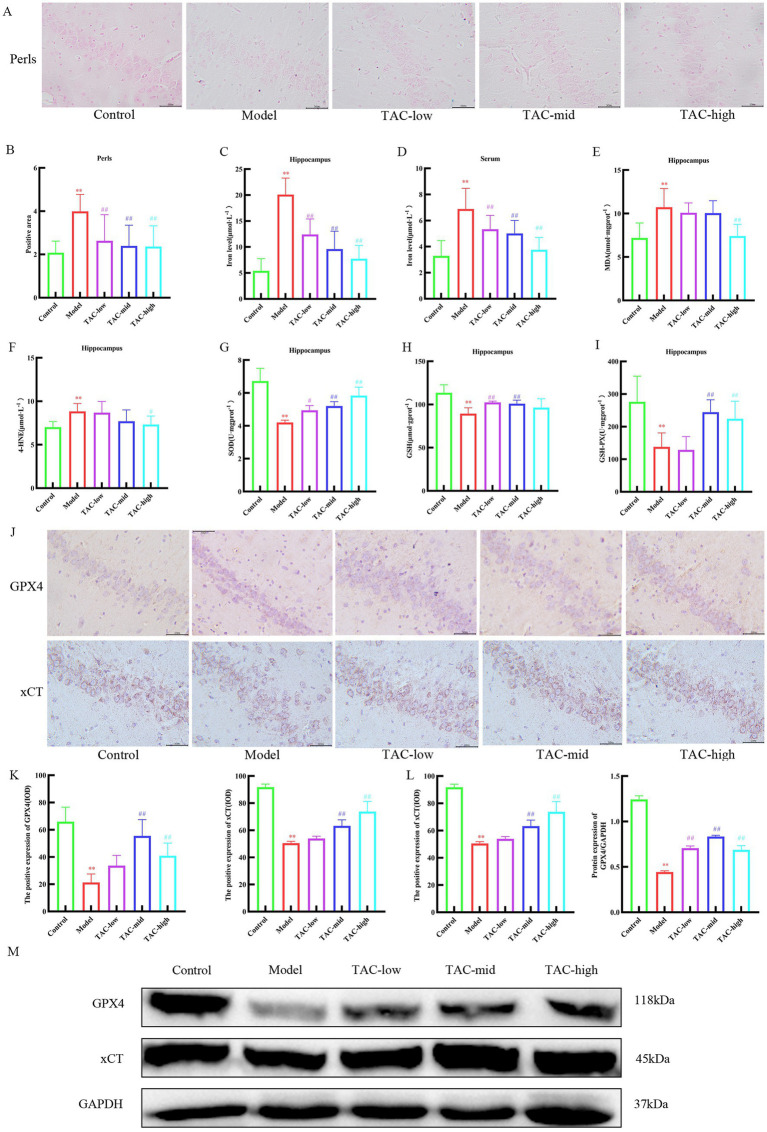
TAC on ferroptosis in the brain tissue of diabetic rats. **(A,B)** Prussian blue staining results of rats in each group. **(C–I)** Effects of TAC on hippocampal tissue iron **(C)**, serum iron **(D)** and SOD **(E)**, MDA **(F)**, 4-HNE **(G)**, GSH **(H)**, and GSH-PX **(I)** in diabetic rats **(J,K)** Results of immunohistochemical detection of GPX4 and xCT in hippocampus. **(L,M)** Results of GPX4 and Xct Western blot detection in hippocampus. Mean ± SD. *n* = 3. And statistical analysis using one-way ANOVA **p < 0.01 vs. Control group; ^#^*p* < 0.05 vs. Model Group. ^##^*p* < 0.01 vs. Model Group.

Prussian blue staining of brain tissue showed that iron deposition increased significantly in Model group, and decreased significantly after TAC treatment (Figures 8A,B). Consistently, the contents of iron (Figures 8C,D), MDA (Figure 8E), and 4-HNE (Figure 8F) were significantly regulated, while the levels of SOD (Figure 8G), GSH (Figure 8H), and GSH-PX (Figure 8I) were significantly upregulated in the brain of diabetic rats given TAC. IHC and WB were used to detect the expression levels of GPX4 and xCT protein in rats (Figures 8J–M). The results showed that TAC could significantly increase the expression levels of GPX4 and xCT protein. Collectively, these results suggest that TAC can reduce the content in the brain of diabetic rats and reduce lipid peroxidation, inhibiting the occurrence of ferroptosis in the brain.

## 4 Discussion

A considerable number of studies have pointed out that the gut microbiota participates in the energy metabolism process and is closely associated with the occurrence and development of diabetes. It is generally acknowledged that excessive dietary nutrition, such as excessive intake of salt, sugar, and fat, exerts a detrimental effect on the diversity and stability of the gut microbial flora, resulting in a decrease in beneficial microbiota and/or an increase in pathogenic microbial communities, inducing chronic low-grade inflammation in the intestine and thereby causing the onset of T2DM (Ma et al., 2019). Simultaneously, the gut microbiota can convey information to the central nervous system via multiple pathways including neural anatomical routes, the endocrine system, the immune system, and metabolism (Needham et al., 2022). The microbiota-gut-brain axis plays a significant role in various neurological disorders, and targeted regulation of the microbiota-gut-brain axis is potentially conducive to the improvement of cognitive impairment (Chen et al., 2017). In studies on T2DM rats with cognitive impairment, it was found that six types of bacteria such as *Firmicutes* (*Lactobacillus* and *Ruminococcus*) and *Bacteroidetes* (*Parabacteroides*, *Bacteroides*, *Butyricimonas*, and *Prevotella*) underwent dynamic alterations at the subspecies level during the occurrence and development of cognitive impairment (Bi et al., 2021). Moreover, experimental studies have demonstrated that restoring the gut microbiota can ameliorate cognitive dysfunction (Hazan, 2020). The results of this experiment demonstrated that the abundance of Bacteroides and Lachnoclotridium in diabetic rats were significantly higher than those in Control group, while that of *Blautia*, *Akkermansia*, and *UCG-005* were significantly lower than those in Control group. Numerous studies have revealed that the abundance of *Lachnoclotridium* in diabetic patients, especially those with type II diabetes, has changed significantly compared to healthy individuals (Goldenberg et al., 2017). This study also provides us with clues about the relationship between bacterial genera, including *Lachnoclostridium* strains, and diabetic cognitive dysfunction.

Recent studies have revealed that traditional Chinese medicines that modulate the microbiota-gut-brain axis possess remarkable potential in alleviating cognitive impairments. For instance, ginsenosides in ginseng and polysaccharides in Dioscorea opposita require the mediation of the gut microbiota to exert biological effects for improving cognitive functions (Bi et al., 2021). Research indicates that the active components of Corydalis have central nervous pharmacological effects such as inhibiting acetylcholinesterase activity (Xiao et al., 2011), activating opioid receptors (Kaserer et al., 2020), and antagonizing dopamine receptors (Zhang et al., 2014). Berberine in the total alkaloids of Corydalis can alleviate anxiety caused by ovariectomy by regulating the gut microbiota (Fang et al., 2021). Moreover, studies have demonstrated that Corydalis can promote insulin release, reduce insulin resistance, and regulate the gut microbiota, thereby improving diabetic complications (Feng et al., 2023). We discovered through the Morris water maze experiment that TAC could significantly improve the cognitive dysfunction of diabetic rats and also proved that TAC could regulate the gut microbiota, verifying the close relationship between the gut microbiota and the central nervous system. In terms of diabetes, after TAC treatment, the fasting blood glucose and serum insulin of diabetic model rats were significantly reduced, consistent with previous research results (Xie et al., 2022). Therefore, we suggest that TAC might improve the cognitive dysfunction of diabetic rats by regulating the gut microbiota. TAC significantly decreased the abundance of Blautia, Akkermansia, UCG-005, and Lachnoclotridium, while the abundance of Bacteroides increased significantly at the same time. Studies have found that the dysregulation of the gut microbiome in patients with Alzheimer’s disease may be due to changes in the genus Bacteroides, which may be related to chronic low-grade inflammation (Vogt et al., 2017).

A considerable amount of evidence indicates that the gut microbiota plays a significant role in the occurrence and persistence of inflammation related to intestinal barrier dysfunction and bacterial translocation (Li et al., 2020). It has been reported that the dysregulation of the gut microbiota can disrupt the immune homeostasis of the intestinal barrier, which can lead to the downregulation of IL-22 expression and insufficient secretion of the antibacterial peptide Reg 3γ by epithelial cells, further damaging the intestinal mucosal permeability (Zong et al., 2020). Moreover, the deficiency of IL-22 or IL-23 can also cause dysbiosis of the gut microbiota in mice with a high-frequency diet (Fatkhullina et al., 2018). The results of this study show that the protein expressions of IL-22, IL-23, and Reg3γ in the colon tissues of diabetic model rats were significantly decreased, and the protein expressions of ZO-1 and occludin1 were also significantly reduced. This indicates that the dysbiosis of the gut microbiota in diabetic model rats disrupts the immune homeostasis of the intestinal barrier, thereby increasing the permeability of the intestinal mucosa.

The immune homeostasis of the intestinal barrier and dysbiosis of the microbiota are associated with central neural inflammation. The damage to the intestinal barrier and intestinal mucus enables external antigens to enter the host from the intestinal lumen. Some studies have found that the abundance of microbiota in the feces of patients with cognitive dysfunction is also highly related to the levels of inflammatory cytokines in the blood of patients (Cattaneo et al., 2017). For example, the intestinal mucosa of patients with Parkinson’s disease shows increased permeability, inflammatory signs, and colonic flora invasion (Parker et al., 2020). The dysregulation of the intestinal microbiota caused by diabetes damages the integrity and permeability of the intestinal mucosal barrier, allowing intestinal microbiota and inflammatory factors to enter the central nervous system through multiple pathways such as blood circulation, generating a neuroinflammatory cascade reaction, thereby damaging brain neurons and leading to cognitive dysfunction. The results of this experiment show that the levels of inflammatory factors IL-1β, IL-6, IL-18, and TNF-α in the blood of diabetic model rats were significantly increased compared with the Control group, while the above indicators in the TAC group were decreased to varying degrees. Therefore, we believe that the improvement effect of TAC on cognitive dysfunction in diabetic model rats is closely related to this pathway. Particularly noteworthy is the NLRP3 inflammasome, which, as an intracellular pattern recognition receptor, is a intracellular multimeric protein complex formed by the combination of apoptosis-associated speck-like protein (ASC) and cysteine-containing aspartate proteolytic enzyme (Caspase-1). The NLRP3 inflammasome can be activated by inflammatory factors. After activation, it cleaves a large number of Caspase-1 precursors to form active Caspase fragments, further promoting the maturation of inflammatory factors such as IL-1β, IL-18, and TNF-α. Simultaneously, the activated Caspase-1 binds to GSDMD and cleaves it. Cytokines are released and secreted through protein pores formed at the N-terminus of GSDMD, accelerating the damage to brain neurons (Meng et al., 2014). Further research results show that the protein expressions of NLRP3, Caspase-1p20, and GSDMD-N in the brain tissue of model group rats were significantly increased, while the protein expressions of NLRP3, Caspase-1p20, and GSDMD-N in the brain tissue of TAC group rats were significantly decreased. This indicates that TAC can effectively inhibit the activation of the NLRP3 signaling pathway in the brain tissue of diabetic model rats and block the process of the central neural inflammatory cascade reaction. Therefore, inflammation may be a key hub connecting the microbiota-gut-brain axis, and regulating the microbiota-gut-brain axis through inflammation is a new therapeutic strategy for cognitive dysfunction in diabetes.

Ferroptosis is a novel concept of cell death characterized by iron overload and lipid peroxidation. In the central nervous system, iron homeostasis plays a vital role in enzyme catalysis, mitochondrial function, myelin formation, and synaptic plasticity. Dysregulation of iron homeostasis can cause oxidative stress and inflammation, leading to nerve cell damage and ultimately resulting in neurological diseases (Tang et al., 2022). Existing studies have proved that ferroptosis is involved in the occurrence and development of cognitive dysfunction in diabetes. Under the combined effects of factors such as oxidative stress, insulin resistance, and inflammatory responses, the intracellular iron accumulation in nerve cells increases (Liu et al., 2024), triggering ferroptosis to damage neurons and thereby causing the occurrence of cognitive dysfunction. In this study, through Prussian blue staining and the detection of Fe2+ in serum tissues, it was found that there was excessive accumulation of Fe2+ in the hippocampus of the model group rats, while the accumulation of Fe2+ in the hippocampus of the TAC group rats was significantly reduced. The influence of the xCT-GSH-GPX4 pathway on ferroptosis has been widely accepted (Dixon et al., 2012). The glutamate/cystine transporter system (system xc−) exchanges intracellular glutamate with extracellular cystine. Cystine is a precursor for glutathione synthesis, which can regulate the ratio of cysteine/glutathione and protect cells from oxidative damage (Tang et al., 2021). GSH is an important cofactor of GPX4 and can promote the reduction of phospholipid hydroperoxides mediated by GPX4 to alcohols, ultimately reducing ROS accumulation and iron-dependent cell death. Our detection of lipid peroxidation, oxidative stress and other indicators, as well as GSH, GSH-PX, xCT, and GPX4 in the hippocampus of rats, also verified the occurrence of ferroptosis in the hippocampus of diabetic rats.

A large number of studies have demonstrated a close relationship between NLRP3 and ferroptosis. The activation of the NLRP3 signaling pathway can facilitate the occurrence and development of ferroptosis. In the AKI model induced by pesticides, the inhibition of ferroptosis can alleviate the activation of the NLRP3 inflammasome (Zhang et al., 2023); in the acute lung injury model caused by sepsis, NLRP3 interacts with ferroptosis (Cao et al., 2022); in the macrophage model induced by PM, it was discovered that NLRP3 inflammasome inhibitors had an inhibitory effect on the increase in intracellular free iron levels and the expression of iron-related proteins. Meanwhile, this experiment proved that the enhanced immune response induced by ferroptosis might trigger an excessive immune response, induce the activation of inflammasomes and pyroptosis of cells, and the increase in inflammatory cytokines caused by the activation of inflammasomes might contribute to the induction of ferroptosis (Beurel et al., 2020). Recently, it has been found that NLRP3 can regulate ferroptosis in LPS-induced S-AKI. The results of RNA sequencing of mouse kidney tissue were combined with the analysis of the FerrDb V2 database, revealing that the absence of NLRP3 can reduce renal ferroptosis, which is also confirmed by the expression of ferroptosis-driven genes and the detection of tissue iron (Li Z. et al., 2023). This study indicates that while NLRP3/Caspase-1 p20/GSDMS-N is significantly upregulated in the model group, xCT/GPX4 is significantly inhibited, resulting in a significant increase in the accumulation of Fe2+ in neurons. The inhibitory effect of TAC on NLRP3 is also manifested in the ferroptosis of nerve cells. NLRP3/Caspase-1 p20/GSDMS-N significantly decreases, xCT/GPX4 expression significantly increases, and lipid peroxidation and oxidative stress responses in hippocampal tissue are also significantly inhibited. In conclusion, ferroptosis and inflammasomes have established a positive feedback loop, influencing each other. This provides a novel mechanism targeting NLRP3 for diseases involving ferroptosis, which holds significant reference value.

Furthermore, a limitation of this study lies in the fact that only one animal model of diabetic cognitive impairment was selected for investigation, and no reverse validation was carried out. Further reverse validation of the application of TAC in diabetic cognitive dysfunction is required, for instance, through intestinal flora transplantation.

## 5 Conclusion

This study investigated the disruption of gut microbiota in diabetes mellitus, leading to damage of the gut mucosal immune barrier and subsequent initiation of gut inflammation spreading to brain tissue, resulting in inflammatory cascade reactions, neuronal ferroptosis, and cognitive dysfunction. Our study found that TAC can improve the integrity and permeability of the gut mucosa by regulating the gut microbiota, restore gut immune homeostasis, inhibit the spread of gut inflammation to the brain, improve the cascade of brain inflammation, and reduce the occurrence of brain tissue ferroptosis, thereby improving cognitive dysfunction in diabetic rats. These results strongly indicate that the gut microbiota may be an effective target for the treatment of cognitive dysfunction in diabetes, and TAC may be an effective candidate for alleviating cognitive dysfunction caused by diabetes.

## Data Availability

The data presented in the study are deposited in the NCBI Sequence Read Archive (SRA) repository, accession number PRJNA1189586.
